# Atom transfer radical polymer-modified paper for improvement in protein fixation in paper-based ELISA

**DOI:** 10.1186/s13065-019-0622-7

**Published:** 2019-08-22

**Authors:** Lu Qi, Aihong Zhang, Yu Wang, Long Liu, Xinghe Wang

**Affiliations:** 1 Phase I Clinical Trial Center, Beijing Shijitan Hospital, Capital Medical University, Beijing, 100038 China; 2 Institute of Chemical Defense, Beijing, 102205 China

**Keywords:** Paper, Enzyme-linked immunosorbent assay, Graphene oxide, Gold nanoparticles, Atom transfer radical polymer

## Abstract

A newly modified paper-based enzyme-linked immunosorbent assay (P-ELISA) was established by immobilizing more proteins on the paper surface through an atom transfer radical polymerization (ATRP) reaction. In addition, introducing graphene oxide (GO) sheets, Au nanoparticles (AuNps) and two primary antibodies (Ab1s) led to signal amplification and cost reduction.

**Electronic supplementary material:**

The online version of this article (10.1186/s13065-019-0622-7) contains supplementary material, which is available to authorized users.

## Introduction

An enzyme-linked immunosorbent assay (ELISA) is an effective and powerful method for protein detection and has been widely used for immunoassays, especially those for detecting and measuring trace biomarkers in complex samples. However, the poor limit of detection (LOD), the need for expensive and large amounts of Ab1s, the requirement for multiple incubation steps and the need for many washing steps have increasingly limited its application. P-ELISA was first proposed in 2010 by Whitesides’ group as a promising platform and has attracted increasing attention due to its simplicity, speediness and low cost. Despite its popularity, unsatisfactory detection is its main disadvantage and has not been solved. The main reason for this issue is that proteins have a low adhesion to paper [1–3].

To solve this problem, several groups have performed many studies and partially solved the problem. The Chen group increased the signal by employing multi-enzyme carbon nanospheres, the Zhao group enhanced the signal by plasma treatment of paper for protein immobilization, and the Dong group increased the sensitivity by high loading of MnO_2_ nanowires on graphene paper [4–7]. Although these new modifications achieved some improvements, the problem of protein immobilization on paper has not been comprehensively solved. In our study, paper was modified by the ATRP reaction, and many hair-like polymeric chains were generated on its surface to combine with a large number of objects to be detected [8, 9]. Untreated paper is very thin and offers few functional groups. Thus, proteins are difficult to attach to paper surfaces, and even attached proteins are easily washed away. In this work, a new and effective protein immobilization method was studied by introducing an ATRP reaction. As a widely used method of aggregation, ATRP can generate many branches on the paper surface and sharply increase the surface area [10, 11]. P-ELISA modified by an ATRP reaction (AP-ELISA) can bind proteins more firmly because of the many polymer chains, and the effect is even more pronounced for small molecules [12, 13].

To further amplify the detection signal, we introduced GO sheets and AuNps. Graphene is a novel, one-atom thick, two-dimensional graphitic carbon system that has the advantages of a unique structure and easy conjugation with proteins without degrading their biological activity [14, 15]. AuNps are an excellent biological carrier because of its high surface-to-volume ratio and wide range of sizes (1 to 200 nm) [16, 17].

## Methods

### Materials

Bovine serum albumin (BSA), 2-(*N*-morpholino)ethanesulfonic acid (MES), *N*-hydroxy-succinimide (NHS), 1-ethyl-3-(3-dimethylaminopropyl) carbodiimide hydrochloride (EDC), 3,3′,5,5′-tetramethylbenzidine (TMB), glutathione *S*-transferase (GST), GST-primary antibody (GST-Ab1), horseradish peroxidase (HRP), alpha-fetoprotein (AFP) and AFP-Ab1 were purchased from Sigma-Aldrich Chemical (Sigma-Aldrich, USA). Whatman No. 1 filter paper (Whatman International, Ltd., England). Natural graphite powder (40 μm in size) was purchased from Qingdao Henglide Graphite Co. Ltd. (Beijing, China). Chloroauric acid (HAuCl_4_·4H_2_O) and trisodium citrate were obtained from Shanghai Reagent Company (Shanghai, China). Deionized water (R > 18 MΩ) used for all experiments was purified by a Millipore purification system (Shanghai, China). AFP was diluted in phosphate-buffered saline (PBS, 0.05 M, pH 7.0, obtained by mixing stock solutions of KH_2_PO_4_, Na_2_HPO_4_ and 0.1 M KCl), and PBST was PBS containing 0.05% (w/v) Tween 20. Blocking buffer solution consisted of a PBS solution supplemented with 2% (w/v) BSA (pH 7.4).

Transmission electron microscopy (TEM) images were taken with an H-9000NAR instrument (Hitachi, Japan). X-ray photoelectron spectroscopy (XPS) measurements were performed on a PHI Quantera scanning X-ray microprobe (ULVAC-PHI, Japan), which used a focused monochromatic aluminium KR X-ray (1486.7 eV) source for excitation and a spherical section analyser. The samples were centrifuged with a Sorvall Legend Micro 17 centrifuge (Thermo Scientific, USA).

### Methods

#### Preparation of the initiator

The use of untreated paper is greatly restricted because of its low surface energy and strong hydrophobicity. Thus, hydrophilic modification by ATRP is necessary before it is used for ELISA [18, 19]. Briefly, the necessary step is preparation of the initiator according to the following protocol. First, 11-mercapto-1-undecanol was dissolved in tetrahydrofuran. Second, pyridine and nitrogen gas were added. Third, 2-bromoiso-butyryl bromide was gently added after the solution cooled. After stirring for 4 h, the mixed liquor was filtered and dried under nitrogen. Finally, the initiator was obtained and stored at 4 °C under nitrogen for use.

#### Preparation of paper-ATRP-protein

The paper was placed in an eppendorf micro test tube (EP tube) containing 500 μL of initiator, and then was placed on a rotating instrument overnight. After washing with methanol, the ATRP reaction mixture (2 M GMA, 0.02 M CuCl, 0.03 M *N*, *N*, *N*′, *N*′,*N*″-pentamethyl diethylenetriamine, and 0.001 M CuCl_2_ dissolved in cyclohexanol) and 0.03 M glucose were added and shaken at RT for 24 h.

1. Ring-opening reaction. A 60% (v/v) ethylenediamine solution was prepared in 50:50 isopropanol/water (v/v). Paper obtained from the above step (modified with high-density epoxy groups) was placed into the ethylenediamine solution at 80 °C for 4 h to expose the amino groups.
2. Modification of aldehyde groups. A 40% (v/v) glutaraldehyde solution was prepared in PBS solution. The paper was placed in the glutaraldehyde solution and incubated at RT for 12 h, producing galdehydes on paper surfaces.
3. Protein loaded onto the paper surfaces. A 2 mg/mL target protein was prepared in PBS. Then, sodium cyanoborohydride was added to the solution to a final concentration of 5 mg/mL. The modified paper was added to the solution and washed using 50 mM Tris–HCl buffer after reaction at 4 °C for 24 h.
4. Sealing side residual aldehydes. The paper was placed in PBS solution containing 1% amino alcohol at 4 °C for 8 h. After washed with 50 mM Tris–HCl buffer three times, the paper-ATRP-protein was obtained.

To investigate the difference in the binding ability of paper to proteins before and after ATRP modification, an amino acid fragment (20–100) from hepatic erythropoietin with a molecular weight of approximately 8.8 kDa was used as a model.

Under the action of the polymer chains, more proteins were firmly attached to the surface of the paper and were not washed off easily because of the blocking action [20–26].

#### Preparation of AuNps and Ab1-AuNps-Ab1′

The AuNps in our research were made using a method similar to the conventional procedure. First, all glassware used in the experiment was thoroughly washed with aqua regia (three parts HCl, one part HNO_3_), rinsed in doubly distilled water, and oven-dried prior to use. Second, 100 mL of 0.01% (mass percentage) HAuCl_4_·4H_2_O in doubly distilled water was brought to a boil under continuous stirring. Then, 2.5 mL of l % (mass percentage) sodium citrate solution was quickly added, stirred, and kept boiling for another 15 min. The solution colour changed from grey to blue, then purple, and finally to wine red during this period. Boiling was sustained for 10 min, the heating source was removed, the suspension was stirred for another 15 min, and it was stored in dark bottles at 4 °C for use.

**Fig. 1:**
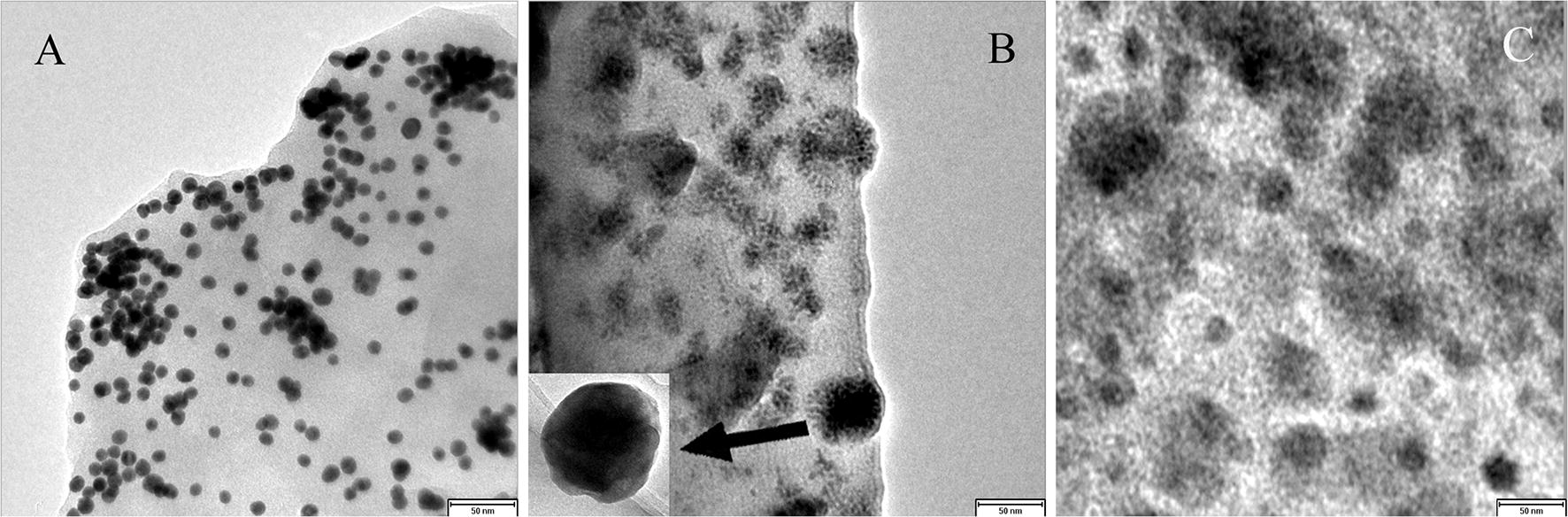
TEM images of AuNps (**a**), Ab1-AuNs-Ab1′ (**b**) bioconjugates and Ab1-AuNps (**c**)

To increase the combination ratio of Ab1 s and reduce the cost, AuNps and double Ab1 s were introduced in this study [27–31]. First, 30 μg of Ab1 and assisted primary antibody (Ab1′, specifically GST-Ab1) were added to a 1 mL suspension of AuNps. Following the literature [32], the molar ratio of Ab1:Ab1′ was 1:10. After incubation at RT for 2 h with gentle stirring, Ab1 and Ab1′ were adsorbed onto the AuNps surface through a combination of interactions. Second, after blocking with BSA, the Ab1-AuNps-Ab1′ complex was centrifuged at 13,300 rpm. Third, after the supernatant was discarded, Ab1-AuNps-Ab1 was obtained at the bottom. Finally, the conjugate was dispersed in PBS containing 1% BSA to increase its stability and minimize nonspecific adsorption during storage at 4 °C. The TEM images of AuNps, Ab1-AuNps and Ab1-AuNps-Ab1′ are displayed in Fig. 1 [33–35].

#### Preparation of Ab2-GO-HRP

**Fig. 2:**
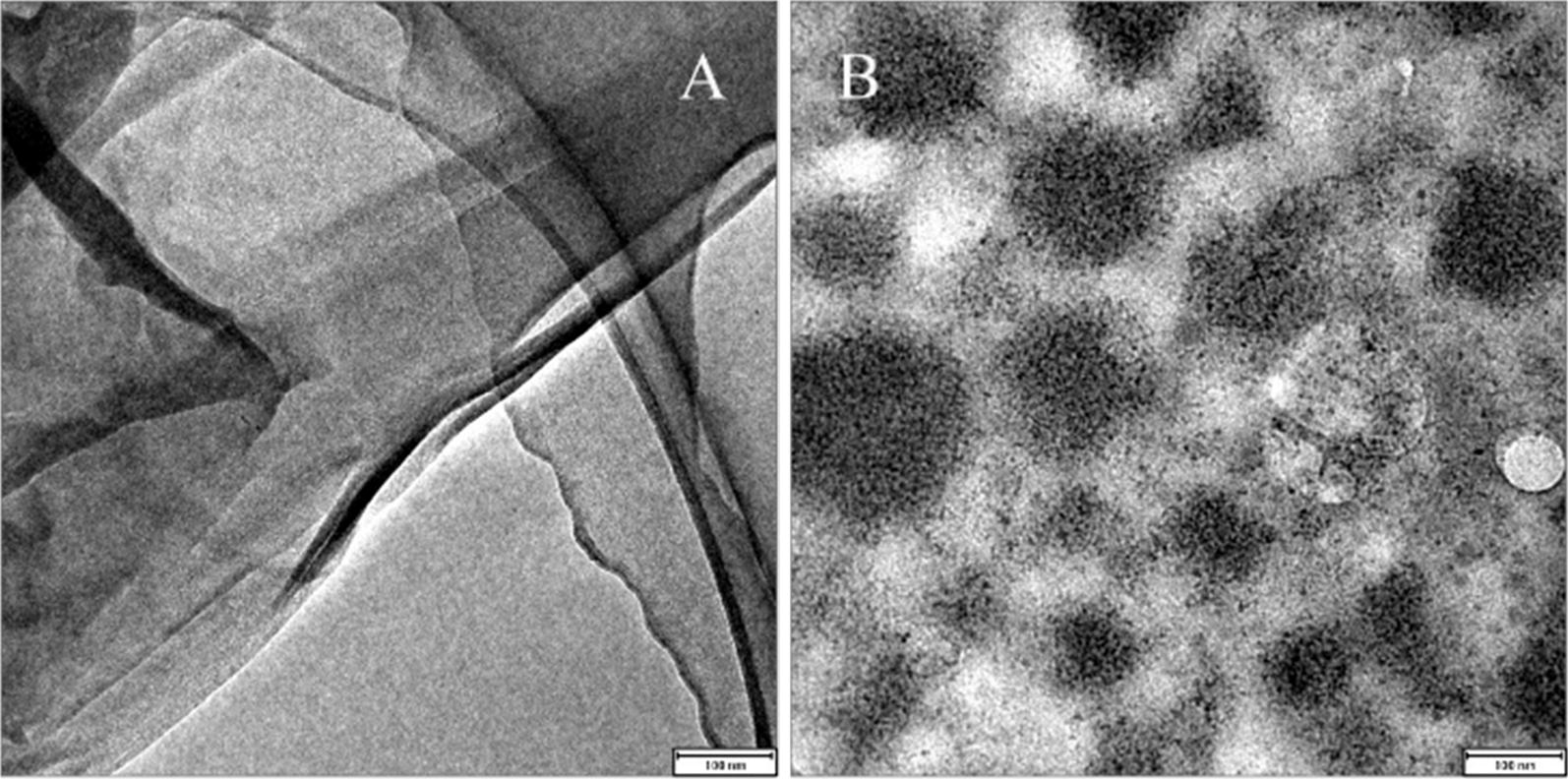
TEM images of the synthesized GO (**a**) and Ab2-GO-HRP (**b**)

The GO used in this study was prepared using a modified Hummers method [36–38]. The secondary antibody-GO-horseradish peroxidase (Ab2-GO-HRP) conjugate was synthesized according to the following protocol. First, 50 mg of ClCH_2_COONa and 50 mg of NaOH were added to a 1 mg mL^−1^ GO suspension. After bath sonication for 1.5 h, the mixed liquor was washed three times. Second, MES buffer containing 400 mM EDC and 200 mM NHS was added. After 30 min of reaction, a homogeneous black suspension was obtained. Third, after washing 3 times, the polymer was suspended in PBS and stirred for 4 h at RT. Finally, Ab2-GO-HRP was resuspended in PBS containing 1% BSA and stored at 4 °C for use. TEM images of GO and Ab2-GO-HRP are displayed in Fig. 2.

To ensure that HRP and Ab2 were successfully bonded to GO, XPS analysis was conducted (shown in Additional file 1: Fig. S1).

The Ab2-GO-HRP spectrum exhibits a single sharp N 1 s peak centred at 399.6 eV (curve A in Additional file 1: Fig. S1), while the spectrum of GO does not (curve B in Additional file 1: Fig. S1). This result indicates that the dark spots originate from Ab2-GO-HRP [4, 39, 40].

## Results and discussion

**Fig. 3:**
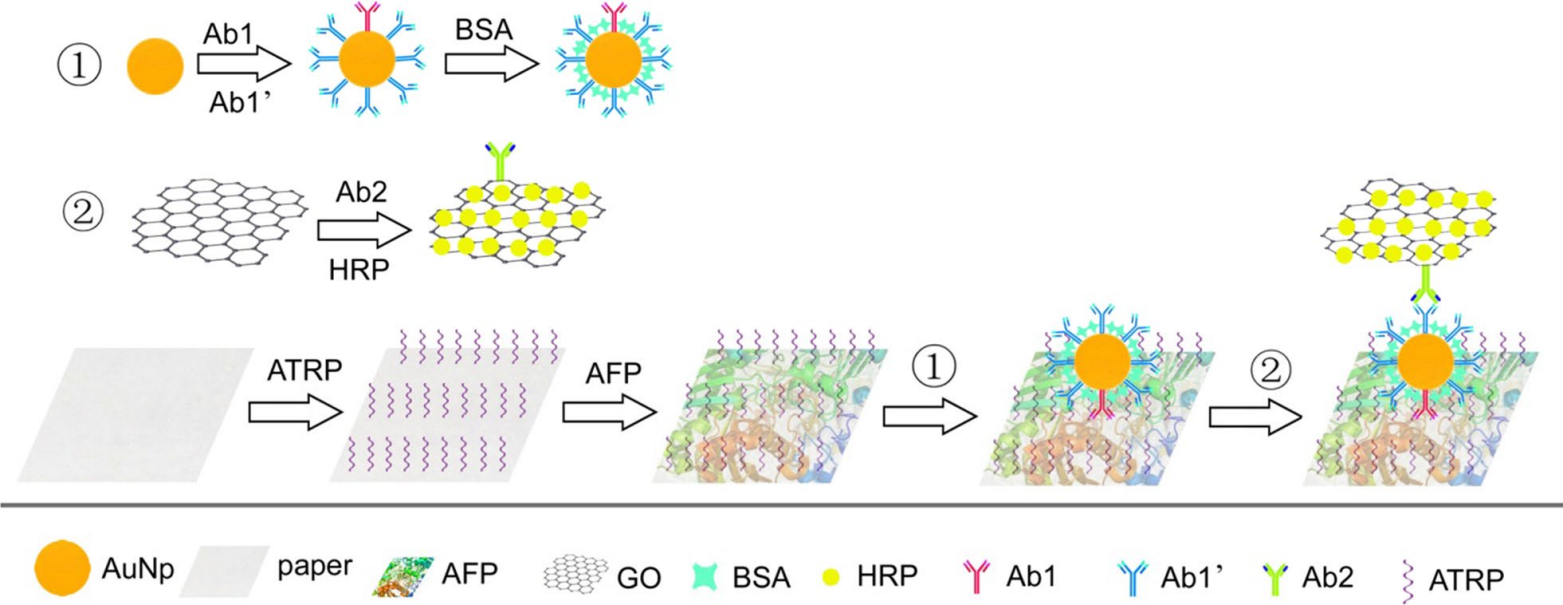
Schematic diagram of the standard AP-ELISA procedure

We successfully modified an AP-ELISA method by introducing an ATRP reaction, AuNps and GO (shown in Fig. 3). Compared with unmodified paper, the ATRP-modified paper demonstrated greater ability to prevent the loss of small molecules and increased the detection signal of the target protein or peptide.

In order to investigate the properties of treated paper, the following experiments were carried out. The results showed that compared with the unmodified paper, the paper modified by ATRP had a stronger binding to protein and exhibited a 70% increase in binding capacity (shown in Table 1) [23–27].

**Table 1:** Comparison of P-ELISA and conventional AP-ELISA

m_1_ (mg)	m_2_ (mg)	Load increase (%)
22.36	38.08	70.30
20.66	36.69	77.59
21.82	36.88	69.02
22.22	39.22	76.51
21.29	37.36	75.48
21.09	36.32	72.21

The load increase of amino acid fragment on the membrane was measured using the following Eq.

1 $$ {\text{Load increase }} = \left( {{\text{m}}_{ 2} - {\text{m}}_{ 1} } \right)/{\text{m}}_{ 1} \times 100\% $$

where m_1_ is the amount of protein adsorbed by raw paper in P-ELISA method, and m_2_ is the amount of protein adsorbed by ATRP modified paper in of AP-ELISA method.

**Fig. 4:**
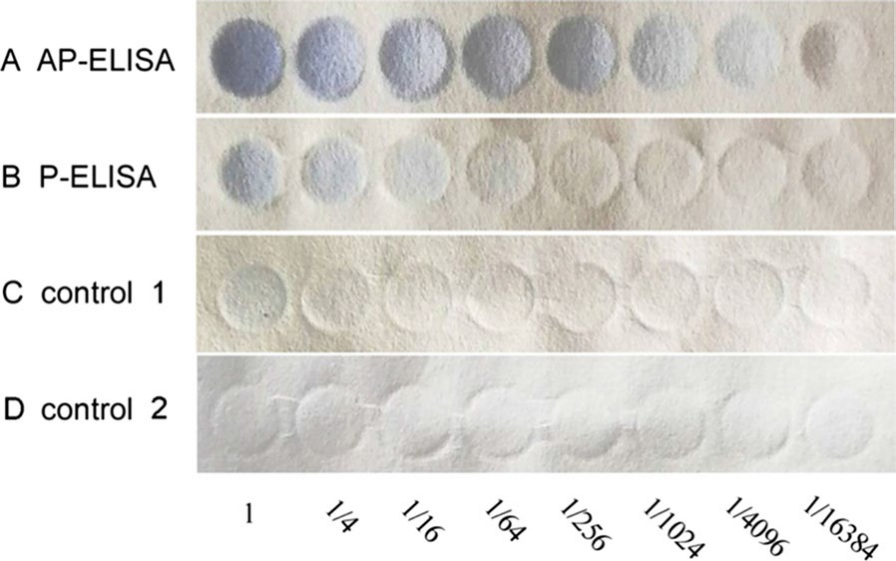
A fourfold dilution series of AFP was detected using AP-ELISA (**a**) and P-ELISA method (**b**). The paper used in control 1 is treated paper and in control 2 is pristine paper

To verify the reliability of the newly developed AP-ELISA method, patient serum samples containing AFP collected from Beijing University Cancer Hospital (Beijing, China) were examined by the AP-ELISA method and the exiting P-ELISA method. The results are shown in Fig. 4.

As shown in Fig. 4, the samples in the vertical columns contained the same amount of AFP, and the samples in the horizontal rows represented a fourfold dilution series. AFP was detected in the first seven columns using the AP-ELISA method, while it was detected in only the first three columns using P-ELISA. Therefore, the modified AP-ELISA strategy was 256 times more sensitive than the P-ELISA method. The calibration curves of AP-ELISA and P-ELISA were shown in Additional file 1: Fig. S2.

**Fig. 5:**
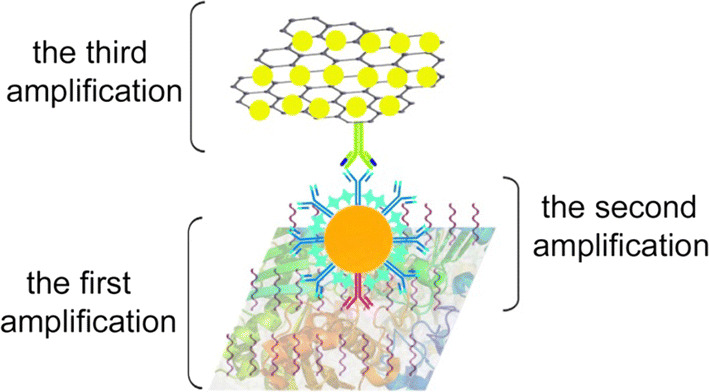
Schematic of the triple signal amplification

The improvement in the LOD for the AP-ELISA strategy is a consequence of the step-by-step signal amplification resulting from introducing ATRP, GO sheets and AuNps [41, 42]. Because the paper has strong adhesion ability after treatment, it can attach more antigens than the untreated paper, which is the first amplification. Then, AuNps conjugate with several copies of Ab1 and Ab1′, which is the second amplification [43]. Finally, GO sheets conjugate more HRP than Ab2, resulting in an increase in the ratio of HRP to Ab2, which is the third amplification (shown in Fig. 5).

Cost reduction is another significant improvement in AP-ELISA over P-ELISA. Low cost is one of the characteristics of this method. In this study, a portion of the expensive Ab1 was replaced by the cheap Ab1′, and this significantly decreased the cost of AP-ELISA. To our knowledge, this report is the first on a modified P-ELISA method that amplifies the signal three times and reduces the cost by introducing two kinds of Ab1.

The advantages of AP-ELISA are shown in Table 2.

**Table 2:** Comparison of AP-ELISA and P-ELISA

	AP-ELISA	P-ELISA
Carrier	ATRP-paper	Paper
Primary antibody	Ab1-AuNps-Ab1′	Ab1
Secondary antibody	Ab2-GO-HRP	Ab2-HRP
Sensitivity	High	Low
Cost	Lower	Low
Prospects	Broad	Narrow

**Fig. 6:**
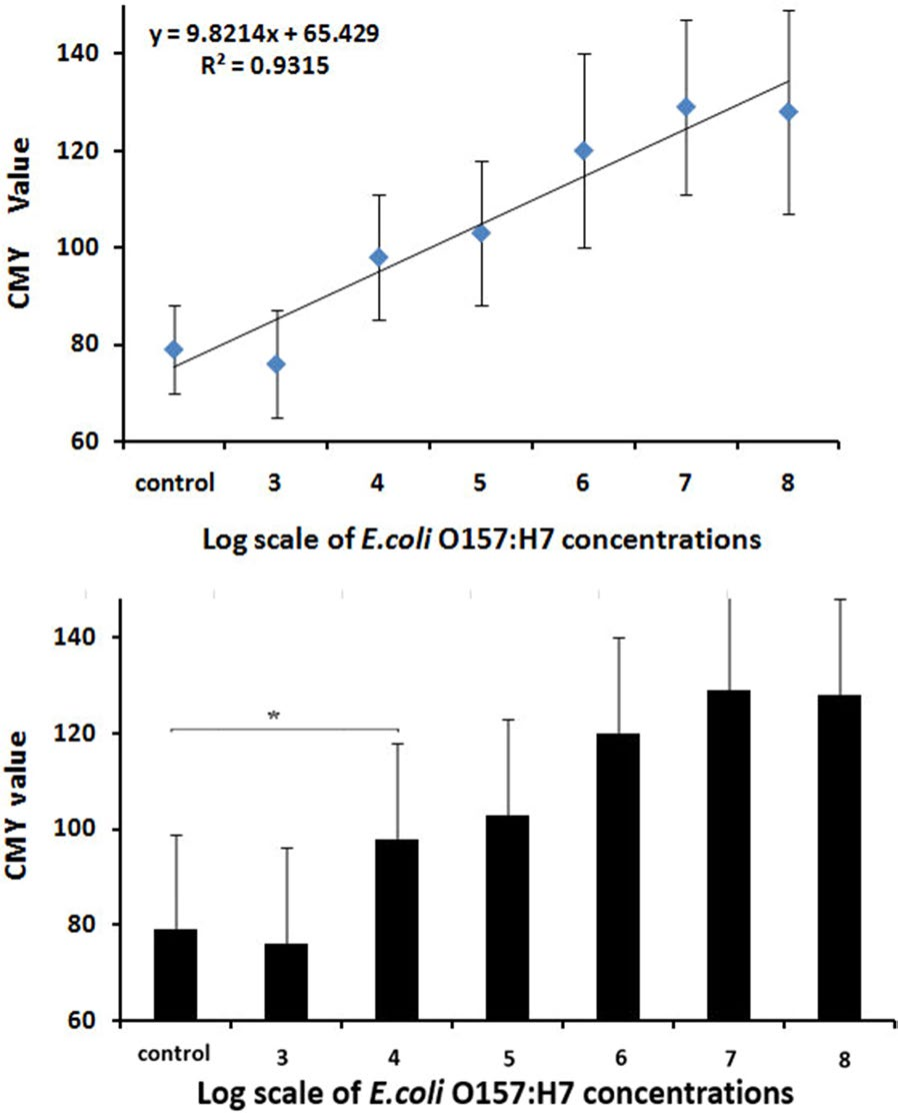
Results indicate that there were significant differences between 1 × 10^3^ CFU/mL *E. coli* O157:H7 and the control group (P < 0.05, asterisk)

*Escherichia coli* serotype O157:H7 (*E. coli* O157:H7) is an epidemic human pathogen responsible for countless deaths [1, 2]. Even now, this situation still exists in undeveloped areas. It is urgent to detect *E. coli* O157:H7 accurately and simply. A total of 0.5 g of lettuce picked from a local garden was treated by grinding, washing and filtering. The concentrations of *E. coli* O157:H7 in successive filtrates were 10^7^, 10^6^, 10^5^, 10^4^, and 10^3^ CFU/mL. The cyan-magenta-yellow (CMY) grey value is expressed as the mean ± standard deviation (${\bar{\text{x}}}$ ± SD). The Shapiro–Wilk test and the Kolmogorov–Smirnov test were used to verify the assumption of normality. Analysis of variance (ANOVA) and *t* test were used, and all analyses used a two-sided 0.05 significance level. The results showed that the standard calibration curve of vegetables was established over the range of 10^3^–10^7^ CFU/mL (shown in Fig. 6).

These findings show that the newly modified AP-ELISA method was effective in complex matrix detection, and the LOD reached 1 × 10^3^ CFU/mL.

## Conclusion

In summary, we successfully developed an ultrasensitive AP-ELISA method with triple signal amplification and cost reduction by introducing ATRP-modified paper, GO sheets, AuNps and double Ab1 s. The results suggest that the AP-ELISA method is feasible for detecting target proteins, especially small molecules. Moreover, compared to the existing P-ELISA method, the AP-ELISA method is 256-fold more sensitive, and the cost is only one-third of the original method. To our knowledge, this report is the first using ATRP as the protein immobilization method for P-ELISA. More importantly, this immobilization strategy can be applied not only to P-ELISA but also to other biological immunoassay methods and biosensors based on the covalent immobilization of protein on paper.

## Additional file

**Additional file 1.** Additional figures.

## Data Availability

Data supporting our findings is contained within the manuscript; any additional data will be shared upon request to the corresponding author.

## Abbreviations

Ab1: primary antibody
Ab1′: assisted primary antibody
Ab2: second antibody
AFP: alpha-fetoprotein
AuNps: Au nanoparticles
ANOVA: analysis of variance
AP-ELISA: P-ELISA modified by an ATRP reaction
ATRP: atom transfer radical polymer
BSA: bovine serum albumin
CMY: cyan-magenta-yellow
EDC: 1-ethyl-3-(3-dimethylaminopropyl) carbodiimide hydrochloride
ELISA: enzyme-linked immunosorbent assay
*E. coli* O157:H7: *Escherichia coli* serotype O157:H7
GO: graphene oxide
GST: glutathione *S*-transferase
HRP: horseradish peroxidase
LOD: limit of detection
MES: 2-(*N*-morpholino) ethanesulfonic acid
NHS: *N*-hydroxysuccinimide
P-ELISA: paper-based ELISA
PBS: phosphate-buffered saline
PBST: phosphate-buffered saline-Tween 20
RT: room temperature
TEM: transmission electron microscopy
TMB: 3,30,5,50-tetramethyl-benzidine
XPS: X-ray photoelectron spectroscopy

## References

[1] Pang B, Zhao C, Li L, Song X, Xu K, Wang J (2018). Development of a low-cost paper-based ELISA method for rapid *Escherichia coli* O157: H7 detection. Anal Biochem.

[2] Chen Y, Chu W, Liu W, Guo X, Jin Y, Li B (2018). Paper-based chemiluminescence immunodevice for the carcinoembryonic antigen by employing multi-enzyme carbon nanosphere signal enhancement. Microchim Acta.

[3] Reis CGR, Angelo HR, Rezende AFS, Brum AA, Azevedo VAC, Borsuk S (2014). Development of an ELISA using the recombinant protein CP1957 of *Corynebacterium pseudotuberculosis* for diagnosis of caseous lymphadenitis in sheep. BMC Proc.

[4] Leivo J, Makala J, Rosenberg J, Lamminmaki U (2016). Development of recombinant antibody-based enzyme-linked immunosorbent assay (ELISA) for the detection of skatole. Anal Biochem.

[5] Dong S, Xi J, Wu Y, Liu H, Fu C, Liu H (2015). High loading MnO_2_ nanowires on graphene paper: facile electrochemical synthesis and use as flexible electrode for tracking hydrogen peroxide secretion in live cells. Anal Chim Acta.

[6] Zhu XX, Xiong SD, Zhang JQ, Zhang XY, Tong X, Kong S (2018). Improving paper-based ELISA performance through covalent immobilization of antibodies. Sens Actuators B.

[7] Nakamura K, Sakuragi N, Ayabe T (2013). A monoclonal antibody-based sandwich enzyme-linked immunosorbent assay for detection of secreted α-defensin. Anal Biochem.

[8] Wang Y, Xu H, Luo J, Liu J, Wang L, Fan Y (2016). A novel label-free microfluidic paper-based immunosensor for highly sensitive electrochemical detection of carcinoembryonic antigen. Biosens Bioelectron.

[9] Chung MK, Regazzoni L, McClean M, Herrick R, Rappaport SM (2013). A sandwich ELISA for measuring benzo[a]pyrene–albumin adducts in human plasma. Anal Biochem.

[10] Preechakasedkit P, Siangproh W, Khongchareonporn N, Ngamrojanavanich N, Chailapakul O (2018). Development of an automated wax-printed paper-based lateral flow device for alpha-fetoprotein enzyme-linked immunosorbent assay. Biosens Bioelectron.

[11] Markwalter CF, Jang IK, Burton RA, Domingo GJ, Wright DW (2017). Biolayer interferometry predicts ELISA performance of monoclonal antibody pairs for *Plasmodium falciparum* histidine-rich protein 2. Anal Biochem.

[12] Gonzalez RM, Zhang Q, Zangar RC, Smith RD, Metz TO (2011). Development of a fibrinogen-specific sandwich enzyme-linked immunosorbent assay microarray assay for distinguishing between blood plasma and serum samples. Anal Biochem.

[13] Shih C-M, Chang C-L, Hsu M-Y, Lin J-Y, Kuan C-M, Wang H-K (2015). Paper-based ELISA to rapidly detect *Escherichia coli*. Talanta.

[14] Oh J, Lee J-H, Koo JC, Choi HR, Lee Y, Kim T (2010). Graphene oxide porous paper from amine-functionalized poly(glycidyl methacrylate)/graphene oxide core-shell microspheres. J Mater Chem.

[15] Gong H, Cradduck M, Cheung L, Olive DM (2012). Development of a near-infrared fluorescence ELISA method using tyramide signal amplification. Anal Biochem.

[16] Huang L, Wang DB, Singh N, Yang F, Gu N, Zhang XE (2018). A dual-signal amplification platform for sensitive fluorescence biosensing of leukemia-derived exosomes. Nanoscale.

[17] Negash M, Kassu A, Amare B, Yismaw G, Moges B (2018). Evaluation of SD BIOLINE *H. pylori* Ag rapid test against double ELISA with SD *H. pylori* Ag ELISA and EZ-STEP *H. pylori* Ag ELISA tests. BMC Clin Pathol.

[18] Kong Q, Wang Y, Zhang L, Xu C, Yu J (2018). Highly sensitive microfluidic paper-based photoelectrochemical sensing platform based on reversible photo-oxidation products and morphology-preferable multi-plate ZnO nanoflowers. Biosens Bioelectron.

[19] Wang Y, Wang Q, Wu A-H, Hao Z-P, Liu X-J (2017). Isolation of a peptide from Ph.D.-C7C phage display library for detection of Cry1Ab. Anal Biochem..

[20] Choi JR, Nilghaz A, Chen L, Chou KC, Lu X (2018). Modification of thread-based microfluidic device with polysiloxanes for the development of a sensitive and selective immunoassay. Sens Actuators B Chem..

[21] Kremer D, Metzger S, Kolb-Bachofen V, Kremer D (2012). Quantitative measurement of genome-wide DNA methylation by a reliable and cost-efficient enzyme-linked immunosorbent assay technique. Anal Biochem.

[22] Güder F, Ainla A, Redston J, Mosadegh B, Glavan A, Martin TJ (2016). Paper-based electrical respiration sensor. Angew Chem Int Ed.

[23] Xu C, Zhang X, Liu X, Liu Y, Hu X, Zhong J (2016). Selection and application of broad-specificity human domain antibody for simultaneous detection of Bt Cry toxins. Anal Biochem.

[24] Zhou M, Yang M, Zhou F (2014). Paper based colorimetric biosensing platform utilizing cross-linked siloxane as probe. Biosens Bioelectron.

[25] Cao L, Fang C, Zeng R, Zhao X, Jiang Y, Chen Z (2017). Paper-based microfluidic devices for electrochemical immunofiltration analysis of human chorionic gonadotropin. Biosens Bioelectron.

[26] Li C-Z, Vandenberg K, Prabhulkar S, Zhu X, Schneper L, Methee K (2011). Paper based point-of-care testing disc for multiplex whole cell bacteria analysis. Biosens Bioelectron.

[27] Wang S, Ge L, Song X, Yu J, Ge S, Huang J (2012). Paper-based chemiluminescence ELISA: lab-on-paper based on chitosan modified paper device and wax-screen-printing. Biosens Bioelectron.

[28] Lei KF, Yang S-I, Tsai S-W, Hsu H-T (2015). Paper-based microfluidic sensing device for label-free immunoassay demonstrated by biotin–avidin binding interaction. Talanta.

[29] Cinti S, Cusenza R, Moscone D, Arduini F (2018). Paper-based synthesis of prussian blue nanoparticles for the development of whole blood glucose electrochemical biosensor. Talanta.

[30] Song Y-Z, Zhang X-X, Ma B, Wu Z-Y, Zhang Z-Q (2017). Performance of electrokinetic stacking enhanced paper-based analytical device with smartphone for fast detection of fluorescent whitening agent. Anal Chim Acta.

[31] Ma L, Nilghaz A, Choi JR, Liu X, Lu X (2018). Rapid detection of clenbuterol in milk using microfluidic paper-based ELISA. Food Chem.

[32] Lin H, Liu Y, Huo J, Zhang A, Pan Y, Bai H (2013). Modified enzyme-linked immunosorbent assay strategy using graphene oxide sheets and gold nanoparticles functionalized with different antibody types. Anal Chem.

[33] Da Silva GO, De Araujo WR, Paixão TRLC (2018). Portable and low-cost colorimetric office paper-based device for phenacetin detection in seized cocaine samples. Talanta.

[34] Ke R, Yang W, Xia X, Xu Y, Li Q (2010). Tandem conjugation of enzyme and antibody on silica nanoparticle for enzyme immunoassay. Anal Biochem.

[35] Sitanurak J, Wangdi N, Sonsa-ard T, Teerasong S, Amornsakchai T, Nacapricha D (2018). Simple and green method for direct quantification of hypochlorite in household bleach with membraneless gas-separation microfluidic paper-based analytical device. Talanta.

[36] Hummers WS, Offeman RE (1958). Preparation of graphitic oxide. J Am Chem Soc.

[37] Song C, Li J, Liu J, Liu Q (2016). Simple sensitive rapid detection of *Escherichia coli* O157:H7 in food samples by label-free immunofluorescence strip sensor. Talanta.

[38] Villarrubia CWN, Soavi F, Santoro C, Arbizzani C, Serov A, Rojas-Carbonell S (2016). Self-feeding paper based biofuel cell/self-powered hybrid μ-supercapacitor integrated system. Biosens Bioelectron.

[39] Verma MS, Tsaloglou M-N, Sisley T, Christodouleas D, Chen A, Milette J (2018). Sliding-strip microfluidic device enables ELISA on paper. Biosens Bioelectron.

[40] Zhang M, Ge L, Ge S, Yan M, Yu J, Huang J (2013). Three-dimensional paper-based electrochemiluminescence device for simultaneous detection of Pb2+ and Hg2+ based on potential-control technique. Biosens Bioelectron.

[41] Gao Y, Deng X, Wen W, Zhang X, Wang S (2017). Ultrasensitive paper based nucleic acid detection realized by three-dimensional DNA-AuNPs network amplification. Biosens Bioelectron.

[42] Fu H, Yang J, Guo L, Nie J, Yin Q, Zhang L (2017). Using the rubik’s cube to directly produce paper analytical devices for quantitative point-of-care aptamer-based assays. Biosens Bioelectron.

[43] Bhardwaj J, Sharma A, Jang J (2019). Vertical flow-based paper immunosensor for rapid electrochemical and colorimetric detection of influenza virus using a different pore size sample pad. Biosens Bioelectron.

