# Supplementary material for: Atom transfer radical polymer-modified paper for improvement in protein fixation in paper-based ELISA
Source: BMC Chem. 2019 Aug 22;13(1):110. doi: 10.1186/s13065-019-0622-7 (PMC6706939; doi:10.1186/s13065-019-0622-7)
Supplement: Supplementary file 1 — Additional file 1. Additional figures. [file 13065_2019_622_MOESM1_ESM.docx]

**Additional file**

**Atom transfer radical polymer-modified paper for improvement in protein fixation in paper-based ELISA**

Lu Qi^a^*, Aihong Zhang^b^, Yu Wang^a^, Long Liu^a^ and Xinghe Wang^a^

^a^Phase I Clinical Trial Center, Beijing Shijitan Hospital, Capital Medical University, Beijing 100038, China

^b^Institute of Chemical Defense, Beijing 102205, China

This Additional file includes additional information as described in the text of the main article:


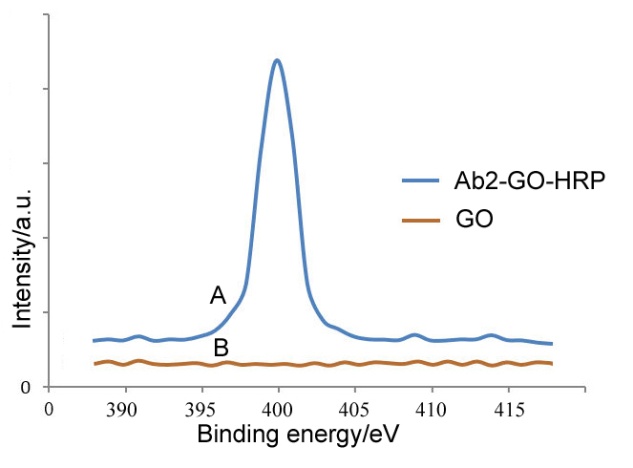

**Fig. S1.** XPS measurements of N1s for Ab2-GO-HRP (A) and GO (B).

The Ab2-GO-HRP spectrum exhibits a single sharp N 1s peak centred at 399.6 eV (curve A in Fig. S1), while the spectrum of GO does not (curve B in Fig. S1). This result indicates that the dark spots originate from Ab2-GO-HRP.


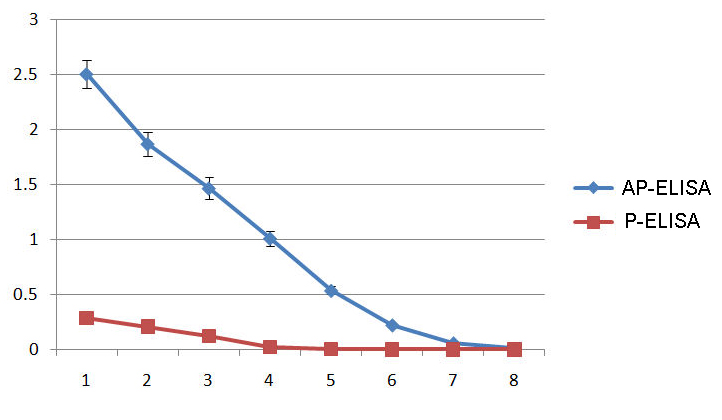


**Fig. S2.** The calibration curves of AP-ELISA and P-ELISA.

AFP was detected using the AP-ELISA and P-ELISA method. Each concentration in the figure was tested six times, and eight different concentrations were detected by two methods. Each concentration represents the average of six independent measurements (N = 6). The error bars represent one standard deviation from the average.
